# Human Deaths and Third-Generation Cephalosporin use in Poultry, Europe

**DOI:** 10.3201/eid.1908.120681

**Published:** 2013-08

**Authors:** Peter Collignon, Frank M. Aarestrup, Rebecca Irwin, Scott McEwen

**Affiliations:** The Canberra Hospital, Garran, Canberra, Australian Capital Territory, Australia (P. Collignon);; Australian National University, Woden, Australian Capital Territory, Australia (P. Collignon);; EU Reference Laboratory for Antimicrobial Resistance. Copenhagen, Denmark (F.M. Aarestrup);; World Health Organization Collaborating Centre for Antimicrobial Resistance in Foodborne Pathogens, Copenhagen (F.M. Aarestrup);; Public Health Agency of Canada. Guelph, Ontario, Canada (R. Irwin);; Ontario Veterinary College/University of Guelph, Guelph (S. McEwen)

**Keywords:** Escherichia coli, E. coli, poultry, antimicrobial resistance, antibiotic resistance, cephalosporin, bacteria, enteric infections

**To the Editor:** Globally, antimicrobial drug resistance is rapidly rising, with resultant increased illness and death. Of particular concern is *Escherichia coli*, the most common bacterium to cause invasive disease in humans (*1*). In Europe, increasing proportions of bloodstream infections caused by *E. coli* are resistant to third-generation cephalosporins (*1*,*2*).

Resistant *E. coli* can be transmitted to humans from animals. A large proportion of resistant isolates causing human infections are derived from food animals (*3*–*6*). However, lack of data has made it difficult to quantify the proportion of antimicrobial drug resistant *E. coli* infecting persons through food sources and the resultant effects on human health. Recent data from the Netherlands now make such estimates possible (*2*,*6*). The additional illness and death among humans resulting from bloodstream infections caused by third-generation cephalosporin–resistant *E. coli* (G3CREC) has been calculated for Europe (*2*). In the Netherlands, there were 205 G3CREC cases during 2007 (4% of all *E. coli* bloodstream infections) (*2*). Another study in the Netherlands revealed that 56% of the resistance genes in G3CREC in humans were identical to genes derived from *E. coli* isolated from retail chicken samples (*6*). Using the findings of Overdevest et al. (*6*) and de Kraker et al. (*2*), we calculated that, in the Netherlands, infections in humans with G3CREC derived from poultry sources were associated with 21 additional deaths. G3CREC-related illness also resulted in 908 hospital bed-days needed to treat persons with these antimicrobial drug resistant bloodstream infections. If these values were extrapolated to all of Europe (i.e., if 56% of G3CREC were derived from poultry), 1,518 additional deaths and an associated increase of 67,236 days of hospital admissions would be counted as a result of cephalosporin and other antimicrobial drug use in poultry (Technical Appendix).

To more accurately estimate the associated increased deaths among persons resulting from third-generation cephalosporin use in poultry, detailed data from more countries is essential. Needed data include records of antimicrobial drug use and resistant bacterial strains found in food animals and domestic and imported foods. However, we already know that G3CREC is rapidly rising in many countries, and in Europe, the infection rate is likely to have tripled from 2007 to 2012 (*2*). Globally, billions of chickens receive third-generation cephalosporins in ovo or as day-old chicks to treat *E. coli* infection, a practice that has resulted in large reservoirs of resistant bacteria. In Canada, this practice has been associated with substantial increases in resistance to third-generation cephalosporins in *Salmonella enterica* serovar Heidelberg isolates detected in humans. (*7*). The United States Food and Drug Administration recently prohibited the off-label use of cephalosporins, including prophylactic uses, in major food animal species, including poultry (*8*).

The number of avoidable deaths and the costs of health care potentially caused by third-generation cephalosporin use in food animals is staggering. Considering those factors, the ongoing use of these antimicrobial drugs in mass therapy and prophylaxis should be urgently examined and stopped, particularly in poultry, not only in Europe, but worldwide.

Technical Appendix Estimated numbers of deaths associated with chicken-derived, third-generation cephalosporin–resistant *Escherichia coli* (G3CREC) bloodstream infections in Europe during 2007 and reported poultry meat consumption in the European Union during 2000–2009.
