# Supplementary material for: Human Deaths and Third-Generation Cephalosporin use in Poultry, Europe
Source: Emerg Infect Dis. 2013 Aug;19(8):1339–40. doi: 10.3201/eid.1908.120681 (PMC3739506; doi:10.3201/eid.1908.120681)
Supplement: Technical Appendix — Estimated numbers of deaths associated with chicken-derived, third-generation cephalosporin–resistant Escherichia coli (G3CREC) bloodstream infections in Europe during 2007 and reported poultry meat consumption in the European Union during 2000–2009. [file 12-0681-Techapp-s1.pdf]

Article DOI: http://dx.doi.org/10.3201/eid1908.120681

Human Deaths and Third-Generation Cephalosporin Use in Poultry, Europe

Technical Appendix

Technical Appendix Table 1. Estimated numbers of deaths associated with chicken derived 3rd generation cephalosporin resistant *Escherichia coli* (G3CREC) blood stream infections In Europe* in 2007†

Country	Population incidence of E. coli blood stream infections per 100,000 population	Population incidence of 3rd Generation cephalosporin SENSITIVE E. coli blood stream infections per 100,000 population	Population incidence of G3CREC (RESIST) blood stream infections per 100,000 population	% G3CREC	No. cases of G3CREC	No. cases of chicken associated G3CREC‡	Excess deaths from G3CREC	No. excess deaths caused by chicken-associated G3CREC‡	Excess no. bed-days from G3CREC (x 1,000)	Excess no. bed-days from chicken-associated G3CREC‡ (x 1,000)
Austria	30	27.3	2.7	9%	226	127	40	22	1.8	1.0
Belgium	19.8	19	0.8	4%	84	47	15	8	0.7	0.4
Bulgaria	7.7	5.9	1.8	23%	139	78	25	14	1.1	0.6
Croatia	27.8	27	0.8	3%	38	21	7	4	0.3	0.2
Cyprus	27.2	21.9	5.3	19%	45	25	8	4	0.4	0.2
Czech Rep.	27.7	25.8	1.9	7%	198	111	35	20	1.6	0.9
Denmark	49.1	47.6	1.5	3%	81	45	14	8	0.6	0.3
Estonia	16.4	16.3	0.1	1%	2	1	-	-	0	0.0
Finland	30.4	29.8	0.6	2%	32	18	6	3	0.3	0.2
France	49.7	48.7	1	2%	617	346	110	62	4.9	2.7
Germany	28.5	26.2	2.3	8%	1,921	1,076	343	192	15.2	8.5
Greece	12.4	11.4	1	8%	110	62	20	11	0.9	0.5
Hungary	11.8	11.2	0.6	5%	59	33	11	6	0.5	0.3
Iceland	34.9	33.6	1.3	4%	4	2	1	1	0	0.0
Ireland	40.7	38.7	2	5%	88	49	16	9	0.7	0.4
Israel	57.4	49.3	8.1	14%	558	312	100	56	4.4	2.5
Italy	17.7	15.7	2	11%	1,149	643	205	115	9.1	5.1
Latvia	6.7	5.7	1	15%	23	13	4	2	0.2	0.1
Lithuania	11.2	10.4	0.8	7%	27	15	5	3	0.2	0.1
Luxembourg	58.9	56.5	2.4	4%	11	6	2	1	0.1	0.1
Malta	28.8	25.1	3.7	13%	15	8	3	2	0.1	0.1
Netherlands	30	28.8	1.2	4%	205	115	37	21	1.6	0.9
Norway	61.2	59.9	1.3	2%	59	33	10	6	0.5	0.3
Poland	7.5	7.3	0.2	3%	64	36	11	6	0.5	0.3
Portugal	40.5	36.4	4.1	10%	431	241	77	43	3.4	1.9
Romania	3.6	2.6	1	28%	225	126	40	22	1.8	1.0
Slovenia	43.2	41.5	1.7	4%	35	20	6	3	0.3	0.2

Country	Population incidence of E. coli blood stream infections per 100,000 population	Population incidence of 3rd Generation cephalosporin SENSITIVE E. coli blood stream infections per 100,000 population	Population incidence of G3CREC (RESIST) blood stream infections per 100,000 population	% G3CREC	No. cases of G3CREC	No. cases of chicken associated G3CREC‡	Excess deaths from G3CREC	No. excess deaths caused by chicken-associated G3CREC‡	Excess no. bed-days from G3CREC (x 1,000)	Excess no. bed-days from chicken-associated G3CREC‡ (x 1,000)
Spain	44.7	41.6	3.1	7%	1,385	776	247	138	10.9	6.1
Sweden	49.1	48.1	1	2%	90	50	16	9	0.7	0.4
Turkey	14.8	8.9	5.9	40%	4,440	2,486	793	444	35.1	19.7
UK	38.8	34.2	4.6	12%	2,821	1,580	504	282	22.3	12.5
Europe (31 countries)	28.2	25.6	2.6	9%	15,183	8,502	2,712	1,519	120.1	67.3

*Countries include European Union member states, excluding Slovakia, both candidate countries (Croatia and Turkey), 2 European Free Trade Association countries (Iceland and Norway), and Israel.
†See (*1*).
‡56% of resistance in G3CREC is derived from poultry (*2*).

Technical Appendix Table 2. Poultry meat consumption in European Union, 2000–2009*

	Food supply quantity (kg/capita/yr)									
Country	2000	2001	2002	2003	2004	2005	2006	2007	2008	2009
Austria	17.1	17.9	17.3	16.8	18	18.3	16.8	17.5	17.1	17.9
Belgium	19.8	22.3	25.6	22.1	22.2	25.6	23.6	25.1	24.4	21.5
Bulgaria	16.9	18.5	21.7	15.8	17.6	18	18.8	19.9	21.3	21.4
Cyprus	29.3	29.7	30.3	28.9	28.9	30.2	24.7	26.8	28.2	26.9
Czech Republic	22.3	24.2	22.9	23.4	25.5	26.4	26.4	24.6	24.7	25.1
Denmark	17.3	20.3	20.2	18.3	16.9	19.7	19.1	20	22.7	18.9
Estonia	17.6	19.7	21.4	21	15.5	16.1	16	17.3	17.8	19.4
Finland	13.7	15.7	17	17.8	18	17.9	17.3	18.8	19.9	18.8
France	26.5	27.7	25.8	24.6	24.4	20.9	20.2	21.1	21.2	22.3
Germany	12.9	14.3	13.3	13.7	14.8	14.8	14.3	15.5	17.1	17.3
Greece	13.3	16.1	15.2	20.6	17	18	14	13.5	13.6	13.7
Hungary	34.1	34	37.1	34.1	34	33.1	28.9	27.5	27.6	26.4
Ireland	30.9	28.1	27.5	27.5	27.8	32.2	28.1	26.1	27.6	26
Italy	18.9	18.3	18	15	15.7	15.4	13.5	15.8	17	17.3
Latvia	10.3	11.8	15.5	16.3	18.4	19.1	20.4	20.5	21	19.5
Lithuania	9.8	11.1	13.2	14.8	20.2	22.6	24.4	24.9	24.9	22.6
Luxembourg	13.8	17.8	15.2	16.8	16.2	15.7	14.4	15.7	17.5	16.6
Malta	15.5	17.7	19	20.7	23.9	21.9	22	24.4	25.1	26
Netherlands	12.6	14.1	12.2	10.9	23	20.6	15	14.4	27.2	22.7
Poland	14.5	17.1	19.6	18.3	19.4	20.4	20.2	21.1	18.5	21.4
Portugal	26.6	28.3	24.6	21.5	22.9	23.8	23.4	26.3	27.6	28.7
Romania	13	15.9	19.4	19.4	19.1	20.7	19	19.3	18.6	21
Slovakia	12.6	12.4	18.2	18.7	18.2	19.9	19.7	18	18	17.4
Slovenia	29	24.1	22.5	23.2	20.6	22.8	20.3	19.9	24.6	26.5
Spain	25.3	26.2	30.2	29.7	26.8	26.9	25.8	27.3	25.3	27.5
Sweden	10.2	11.2	13.2	12.7	12.9	14	13.9	14.8	16.1	15.4
United Kingdom	28.5	29.2	29.1	29.8	31	30.8	30.8	29.1	28.2	32.2

*Data from (3). EU human poultry consumption from FAOSTAT, the FAO statistical database (http://faostat.fao.org/?lang=en).

References

1. de Kraker ME, Davey PG, Grundmann H; BURDEN study group. Mortality and hospital stay associated with resistant *Staphylococcus aureus* and *Escherichia coli* bacteremia: estimating the burden of antibiotic resistance in Europe. PLoS Med. 2011;8:e1001104. Epub 2011 Oct 11. PubMed http://dx.doi.org/10.1371/journal.pmed.1001104

2. Overdevest I, Willemsen I, Rijnsburger M, Eustace A, Xu L, Hawkey P, et al. Extended-spectrum β-lactamase genes of *Escherichia coli* in chicken meat and humans, the Netherlands. Emerg Infect Dis. 2011;17:1216–22. PubMed http://dx.doi.org/10.3201/eid1707.110209

3. US Food and Drug Administration. Cephalosporin order of prohibition goes into effect. April 6, 2012 [cited 2012 Apr 20]. http://www.fda.gov/AnimalVeterinary/NewsEvents/CVMUpdates/ucm299054.htm
